# Hygienic Rules for the Preservation of Health in Western Africa and Other Tropical Climates

**Published:** 1855

**Authors:** William F. Daniell

**Affiliations:** Staff Surgeon Second Class, &c.


### HYGIENIC RULES FOR THE PRESERVATION OF HEALTH IN WESTERN AFRICA AND OTHER TROPICAL CLIMATES.

By William F. Daniell, M.D., F.R.G.S., Staff Surgeon Second Class, &c.

The preservation of health is under ordinary circumstances a subject of serious consideration; but in unhealthy climates it becomes one of paramount importance. Diseases of tropical countries differ widely from those common to colder regions, and, in general, have a less fortunate issue. Engendered from a multiplicity of sources, they often assume an asthenic or typhoid form, run a sharp and rapid career, and, if unchecked, cease with a melancholy termination. In the alluvial and swampy districts of Western Africa, and even in the more elevated table-lands, the maladies from which Europeans suffer are those generated by what has been termed *malaria*, an endemic or epidemic poison, resulting from a combination of various local and atmospheric influences. In many instances, when concentrated, these influences induce that morbid condition of the body which only ends with life ; and therefore, as the diseases proceeding from these causes are not unfrequently developed in an aggravated form, and a low insidious type, an active and energetic mode of treatment becomes immediately requisite. The climatorial agencies peculiar to the maritime countries of equinoctial Africa have been considered to be more pernicious and unhealthy than those emanating from other portions of the same continent. Their nature and predominant characteristics have not hitherto been satisfactorily determined ; they are nevertheless sufficiently deleterious to create an enormous sacrifice of human life; and, as experience demonstrates, they demand a larger amount of skill and knowledge to combat their depressing action on the frame than similar agencies elsewhere within the tropics. The necessity for the adoption by unacclimated persons of certain prophylactic measures, so as to enable them to withstand the attacks of the local affections, and modify their severity, is too obviously enforced to require comment. There can be little doubt that, by an efficient and judicious code of sanitary regulations, a tolerable state of health may be maintained in several of these pestilential localities for a considerable period; that though such regulations will prove unable to avert the accession of sickness, they will go far towards restraining its violence ; and that, when the ordinary febrile and dysenteric diseases are somewhat divested of their more formidable symptoms, they will have lost much of their intractable character, and consequently will be rendered more amenable to the remedial effects of medicines.

The seasons, on the coast of Africa, may be divided into two, viz., rainy and dry, the former of which is the most unhealthy for white residents and voyagers. The African climate derives its peculiar constitution from excessive atmospheric humidity, conjoined with an elevated temperature, and the constant exhalation of local miasms, more or less influenced in their noxious prevalence and origin by various electrical laws at present unknown and unappreciated. The following rules have chiefly guided the author, during a seventeen years' tour of service in most of the countries of Western Africa; and he has hitherto found them sufficiently effective to aid in the maintenance of good health, and the probable reduction of an otherwise heavy mortality.

1. The dress should be usually constituted of flannel, cotton, or such woollen fabrics as are best adapted for resisting the alternating vicissitudes of heat and cold. Flannel should be invariably worn next the skin, as during the rains it reduces the chilling influence of an active humidity, and in the dry and hot season absorbs the copious perspiration then exhaled, and, by the moistened medium it affords, prevents direct evaporation from the cutaneous surface, a result always to be dreaded in tropical regions. In the cold and rainy season, the shirts, trousers, and, in fact, the entire dress, if composed of flannel, would be found to be the best means for resisting the injurious action of a humid atmosphere or heavy showers, and preventing the occurrence of rheumatism and fever. The head cannot be too carefully protected from a torrid sun; and thick felt caps, or straw hats, well lined, will prove most serviceable. In their selection, those of a black colour should be avoided, as they absorb a greater degree of heat. The feet must also be kept well dried; and woollen socks are preferable to cotton, if they do not produce inconvenience.

2. Personal cleanliness is, in every country, essentially conducive to the preservation of health, but in tropical climates it becomes one of the most important and necessary measures. Ablutions of the body should be performed at least daily, whenever the skin is not too much heated, and where suitable opportunities offer. With reference to cold baths, some precautions are requisite; as a general rule, they ought never to be used by debilitated persons, convalescents, or those who are subject to special visceral diseases, nor whenever the body is bathed in a profuse sweat from labour or exercise. If any chill or sensation of coldness is experienced after their employment, they must be abandoned, and tepid water substituted, which, for the unacclimated visitor, would, on the whole, be found far preferable. Alluvial and muddy water from creeks, rivers, lagoons, and the embouchures of streams, or in the immediate vicinity of the coast, should never be resorted to for bathing purposes, if water of a purer or better quality can be elsewhere obtained. Filtration would remove, to a certain extent, the impurities that abound in fresh water. The surface of the skin, immediately after every bath, should be thoroughly dried by friction with a coarse towel, if no prickly heat or other cutaneous irritation exist. When the body or clothes have been thoroughly wet and saturated, either from accidental circumstances, as from immersions, or long continued exposure to heavy rains, the garments should be first removed without delay, and the skin be well rubbed with strong spirits (rum, schiedam, or *aguardiente*, would answer), until a sensation of warmth and dryness is induced. Dry clothes may then be put on.

3. Undue exposure to the sun should be always avoided; and great care is equally necessary not to create excessive fatigue, if so exposed. The solar heat attains its maximum from $12^h$ to $3^h$ p.m.; all physical exertions or occupations should therefore be considerably restricted within these hours. Rest from all kinds of labour during mid-day will be attended by invigorating results. Business or pleasure avocations at any distance should be deferred until the cooler hours near sunrise or sunset. Whenever imperative duties demand noon-day visits from ship to shore, or from house to house, a cotton or silk umbrella must be employed to shield the head and eyes from the oppressive glare and heat that ordinarily prevail. From $5^h$ 30′ to $6^h$ a.m. is the hour best adapted for rising. Previous, however, to the commencement of the toils of the day, a cup of coffee, tea, or chocolate, should be swallowed, in conjunction with a small piece of bread or biscuit. The intervals least unfavourable for travelling, shooting, or scientific excursions, visits, or general recreations, are from $6^h$ to $9^h$ a.m., and from $4^h$ to $7^h$ p.m., the coolest portions of the day. The time for repose varies from $9^h$ to $11^h$ p.m., or according to the previous habits or inclination of each individual. Moderate horse exercise is preferable to walking, whenever attainable. The propriety of issuing stringent regulations to prohibit all persons from sleeping without proper shelter, and thereby exposed to the heavy night dews, and cold, baneful land winds, however pleasant and refreshing they may be, cannot be too forcibly inculcated; for the surface of the skin, relaxed by constant exudations of moisture, is in that languid condition which renders it more susceptible to morbific influences, and dysenteries or febrile affections sooner or later inevitably follow. Reclining on the damp ground, after protracted exercise, or sleeping in apartments on shore, with open windows, permeated by irregular currents of air either from inland or evening sea breezes, or in houses in close proximity to swampy localities or the banks of rivers, must, whenever practicable, be avoided. The more elevated the quarters are from the ground, the greater will be the degree of salubrity, and the less danger will be produced by prejudicial exhalations from decomposed vegetable matter, or other refuse of the soil.

4. The acclimatisation of European constitutions to the exigencies and vicissitudes of a tropical clime, principally depends on a partial change or adaptation of diet. That the permanency of good health is directly influenced by a normal state of the digestive organs, there can be no question: and the golden rule must therefore be, to use the kind of food best calculated to afford due support to the body, without inducing subsequent exhaustion or debility by impairing the digestive functions, or increasing their activity. The means hitherto discovered most efficient for the accomplishment of this object have been derived from the experience of aboriginal authorities. The habits and customs of the native tribes will frequently be found, in these respects, a tolerably safe guide; proper allowance being made for the moral and physical distinctions of race, previous mode of living, &c. It must ever be borne in mind, that in hot countries comparatively less food is required than in cold ones; and that the stomach, if food be taken at irregular intervals, loses much of its assimilative powers. No precise system of dietetics can be enunciated that would conclusively embrace the comprehensive details of this subject; yet it may be stated as an ordinary maxim, that edibles most nutritious and wholesome, the indigenous products of the place, ascertained to be commonly in vogue, and prepared by the simple processes of native cookery, should more or less enter into the composition of the chief daily meals, the number of which must be regulated in accordance with the necessities of climate and constitution. Animal substances should invariably be well cooked; boiled meat, in the form of soups and stews, is therefore preferable to the same when roasted. More of vegetable, and less of the animal constituents, should be the fundamental principle in the disposition of meals. In tropical Africa, rice, yams sweet potatoes, kous-kous, cocos, and young cassada roots, constitute excellent substitutes for European vegetables.

The oleaginous and other dishes of the natives, composed mostly of mutton, fowls, game, fresh and dried fish, with palm oil, ochros, peppers, yams, molochias, plantains, and other esculents, are very palatable and nourishing, and appear well adapted for European use, if visitors could only be induced to tolerate their somewhat nauseating aspect. Poultry is usually cheap and plentiful; and no better article of food could be purchased. Fish of various kinds, both dried and cured, with shrimps, crawfish, mangrove oysters, etc., can sometimes be obtained in large quantities; when fresh, they are in general of good flavour, and easy of digestion. Oranges, pineapples, guava, melons, limes, bananas, mangoes, papaws, sourtops, custard apples, and other fruits, if immoderately eaten, tend to promote indigestion and diarrhœa, with other irritations of the bowels, and occasionally leading to more serious disease. When desirable, a limited allowance should be taken early in the morning, previous to breakfast. Punctuality in meals, with a certain amount of rest after each, ought, as far as convenient, to be adopted, to facilitate the digestive process.

The judicious use of wine and spirits may be advocated, since they are not detrimental to health, and their daily enjoyment often materially contributes to the alleviation of the climatic languor and debility resulting from an elevated temperature. Good sherry, madeira, and bucellas, may be administered with advantage to invalids and others; and a few glasses at meal times are productive of an appropriate stimulus to the stomach. Port wine is commonly too much adulterated by spirituous compounds and other noxious ingredients to be of benefit. Weak brandy and water may be drunk in moderation throughout the day by those more habituated to the use of ardent spirits, and will in some degree mitigate the continual state of thirst. Claret (Bourdeaux wine) and water is also a pleasant and cooling beverage to persons inclined for weaker potations, and not predisposed to visceral relaxations, dysenteric, and other gastroenteritic maladies. Abstinence from *aguardiente* and other sorts of inferior rum and spirits cannot be too strictly enjoined, as they not only disorder the digestive organs, but exert an injurious effect on the system. Malt liquors should likewise be drunk with some caution, as they tend to produce a bilious and plethoric habit of body, and ultimately dispose to apoplectic and congestive diseases. Allsopp's or Bass's pale ale are, on the whole, preferable to bottled porter, stout, or the stronger ales, as they are manufactured of a greater purity, are milder stimulants, and are less subject to unwholesome adulteration. Occasionally, they have been known to exert a remarkably tonic influence on the worn out and debilitated constitutions of the older colonists and traders. The moderate indulgence of the passions is not prejudicial to health; but great care is requisite that their gratification become not habitual, so as to degenerate into excess, which is too frequently the case with residents in tropical countries.

5. Among the most frequent predisposing causes of sickness, are the dread and prostration of spirits that pervade almost every class of people on their first visit to this unhealthy coast. The unremitting fatality of diseases, united with the depressing influences of climate, have certainly gained for this part of the globe an unenviable notoriety, which time can never dissipate. Notwithstanding the array of fearful drawbacks, residents in the majority of these regions may preserve their health unimpaired for a considerable number of years, by proper care and attention to hygienic considerations, by cheerfulness and confidence relative to future results, by regularity, and an adaptation of diet to the climate, by a determination to resist hypochondriacal forebodings, or despondent impressious, by the appropriate employment of time in judicious mental and physical labour or recreations, and by tranquillity of mind, and an implicit reliance on the ever constant protection of an all-wise Providence. By a conformity to these hints, I have known many Europeans to live in some of the most sickly countries of Western Africa for periods of twenty and thirty years, and even upwards.

[The author of these observations, a military medical officer, having resided for a considerable number of years in many of the most sickly countries of Western Africa, has been enabled to draw out hygienic regulations, based exclusively on the results of his experience, for the guidance of all Europeans who may frequent these tropical regions. We may observe that these regulations were chiefly intended for the use of the mercantile marine, and for persons engaged in commercial enterprises in unhealthy climates. They will subsequently appear in a hydrographical work on Western Africa, by Mr. Findlay, now on the eve of publication. It is our sincere wish that Dr. Daniell's observations may prove of sufficient utility to lessen the fearful mortality that has hitherto occurred among the white residents of our colonial possessions in Africa and elsewhere. At some future date we hope to publish in this Journal a continuation of these rules, referring to medical and direct prophylactic measures. —EDITOR.]

---

### ON THE SCIENTIFIC INVESTIGATION OF SANITARY QUESTIONS.

